# Evidence map of knowledge translation strategies, outcomes, facilitators and barriers in African health systems

**DOI:** 10.1186/s12961-019-0419-0

**Published:** 2019-02-07

**Authors:** Amanda Edwards, Virginia Zweigenthal, Jill Olivier

**Affiliations:** 1 School of Public Health and Family Medicine, Faculty of Health Sciences, University of Cape Town, Anzio Road, Observatory, Cape Town, 7925 South Africa; 2 Western Cape Government Health, Cape Town, South Africa

**Keywords:** Knowledge translation, knowledge translation strategies, health policy, policy-making, mapping review, evidence map, African health systems

## Abstract

**Background:**

The need for research-based knowledge to inform health policy formulation and implementation is a chronic global concern impacting health systems functioning and impeding the provision of quality healthcare for all. This paper provides a systematic overview of the literature on knowledge translation (KT) strategies employed by health system researchers and policy-makers in African countries.

**Methods:**

Evidence mapping methodology was adapted from the social and health sciences literature and used to generate a schema of KT strategies, outcomes, facilitators and barriers. Four reference databases were searched using defined criteria. Studies were screened and a searchable database containing 62 eligible studies was compiled using Microsoft Access. Frequency and thematic analysis were used to report study characteristics and to establish the final evidence map. Focus was placed on KT in policy formulation processes in order to better manage the diversity of available literature.

**Results:**

The KT literature in African countries is widely distributed, problematically diverse and growing. Significant disparities exist between reports on KT in different countries, and there are many settings without published evidence of local KT characteristics. Commonly reported KT strategies include policy briefs, capacity-building workshops and policy dialogues. Barriers affecting researchers and policy-makers include insufficient skills and capacity to conduct KT activities, time constraints and a lack of resources. Availability of quality locally relevant research was the most reported facilitator. Limited KT outcomes reflect persisting difficulties in outcome identification and reporting.

**Conclusion:**

This study has identified substantial geographical gaps in knowledge and evidenced the need to boost local research capacities on KT practices in low- and middle-income countries. Evidence mapping is also shown to be a useful approach that can assist local decision-making to enhance KT in policy and practice.

**Electronic supplementary material:**

The online version of this article (10.1186/s12961-019-0419-0) contains supplementary material, which is available to authorized users.

## Background

The need for research-based knowledge to inform health policy and practice is a chronic global public health concern [1–3]. Knowledge generated through health research has the potential to improve health outcomes, promote service delivery and strengthen health systems functioning [4–6]. However, a consistent finding from the health services literature has been the failure to translate research findings into health policy and practice [7]. Despite burgeoning interest in this know–do gap, the translation process remains slow, haphazard and unpredictable, resulting in reduced health gains vis-á-vis societies’ investment in research [8, 9]. In low-resource, high-disease settings, such as those found in many African countries, the consequences of ineffective knowledge translation (KT) are amplified, emphasising the need for health system decision-makers to justify their decisions based on high-quality evidence [3, 10, 11].

KT is a term commonly used to describe the complex process of moving research-based evidence into policy and practice, although the term (and its related field) is diverse and diffused [12–14]. In this review, our understanding of KT aligns with the Canadian Institute of Health Research, which defines KT as “*a dynamic and iterative process that includes the synthesis, dissemination, exchange and ethically sound application of knowledge to improve health, provide more effective health services and products, and strengthen the health care system*” ([15], p. 1). Within this conceptualisation, ‘knowledge exchange’, ‘knowledge interaction’ and, more recently, ‘integrated knowledge translation’ have also gained traction as these terms emphasise the central role of knowledge users and their influence on the KT process [16–20]. For the purposes of this article, KT is used as the overarching term and these related concepts viewed as implicit in the broader definition.

In recent years, efforts to increase the uptake of health research into policy have intensified globally, resulting in a growing body of literature on KT [16, 21–27]. From this literature, several factors have been found to influence the use of research in policy-making. In an updated systematic review, Oliver et al. [28] identified several barriers and facilitators to evidence uptake by policy-makers. The most frequently reported barriers indicate that poor access to good quality, relevant research and a lack of timely research output greatly decrease the potential for research to influence policy, while collaborations between researchers and policy-makers, skills-building with policy-makers and improved relationships tend to enhance research use. A review by Oliver et al. [28] focused on policy-making across different areas, including criminal justice, education and food policy; however, 126 of 145 included studies related specifically to health policy-making. Focusing on health policy alone, Lavis et al. [29] further emphasised the importance of engaging with research users to enhance the uptake of evidence in health policy decision-making. Oliver et al. [28] go on to describe the importance of informal knowledge in decision-making, such as personal experience, clinical expertise and other tacit-based knowledge. These authors conclude that formal research-based knowledge is just one source of information for policy-makers and that identifying different types of knowledge is a crucial step in getting research to influence policy, a finding increasingly supported by others [5, 17, 30, 31]. Additionally, research institutions that demonstrate capacity for generating high quality, reputable research and close connections with policy-makers have been shown to have greater embeddedness in the policy-making process and therefore a greater influence in translating their research into policy [26, 27, 32].

While various strategies such as policy briefs, collaborative workshops and KT platforms have been proposed to enhance KT [1, 16, 29, 33–36], evidence of their comparative effectiveness remains limited [5, 7, 37]. Possible reasons for this include difficulties identifying what outcomes should be measured, the need to strengthen the validity of measurement instruments and the inherently complex ways in which KT can occur [14, 30, 38]. A key criticism from the literature has been the failure of current KT strategies to account for this complexity, including the specificity of the policy-making process, its power dynamics, differing timelines and unique contextual considerations, especially in low- and middle-income countries (LMICs) [17, 39–41]. These issues make the selection of appropriate, contextually relevant KT strategies difficult for researchers and policy-makers [42]. Moreover, researchers have highlighted the weak relationship between health systems and research systems, recognising the need to consider complex systems issues and their influence on the adoption of new knowledge [43, 44]. Box 1 summarises the central challenges to KT in LMIC settings identified by the current literature.

In Africa, where more than 50% of the world’s LMICs are located, there remains a paucity of research on KT strategy selection and activities that promote the use of research by health policy-makers, particularly in policy formulation processes [42, 45]. Exacerbated by limited funding availability and institutional research capacity to generate locally relevant research [11, 44], there is an urgent need to seek out cross-country learning opportunities that boost understanding of KT strategies in this context.

This paper provides a mapping review of the literature on KT strategies (particularly in the policy formulation process) and their reported outcomes, barriers and facilitators in African health systems, with an emphasis on southern African countries. This paper has four main aims. Firstly, to position the current available research on KT in African health policy-making within the broader field of KT. Secondly, to provide a summarised and synthesised understanding of the current status and key issues within a massive and diverse body of literature for African health system researchers and policy-makers. Thirdly, to identify gaps in current knowledge and areas for potential future research on local KT systems in Africa. Finally, to generate a user-friendly evidence map, testing the usefulness of the evidence-mapping approach for this type of diverse and dispersed topic and for the field of health policy and systems research more broadly.

## Methods: systematic evidence mapping

Evidence mapping is an emerging method of synthesis that provides a systematic overview of the literature on a specific topic [46]. Like full systematic reviews, evidence mapping employs methods that are reproducible and transparent, applying explicit search procedures and robust inclusion/exclusion criteria [47, 48]. However, while systematic reviews target specific research questions, evidence mapping focuses on the nature, volume and characteristics of the literature in order to identify, describe and categorise what is known [49]. Despite methodological similarities, evidence mapping has been distinguished from the scoping review methodology by engaging with stakeholders early in the research process, through the increased rigor of systematic online database searches and by the final production of a visual or searchable database and/or user-friendly ‘map’ [50]. Evidence mapping is particularly useful for synthesising and increasing coherence, giving shape within a broad or diverse field of interest, where information is found in different sectors, and definitions are not concretised (as KT is known to be).

**Fig. 1:**
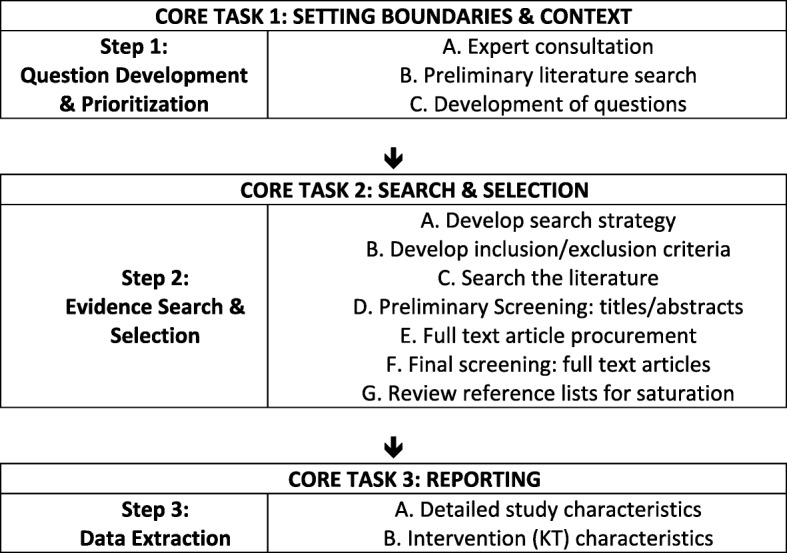
Global evidence mapping method (Adapted from Bragge et al. [49])

For the purposes of this paper, a systematic evidence mapping process was adapted from the Global Evidence Mapping Initiative [49]. Figure 1 outlines this process and the three core tasks involved, including (1) setting the boundaries and contexts of the map; (2) searching and selecting relevant studies; and (3) reporting on yield and study characteristics. What is presented here forms the evidence map of research on KT strategies across African health systems (with a focus on policy formulation).

The three-step mapping process was conducted between July and October 2017. An overarching research question seeking to elucidate what KT strategies are employed by health researchers and policy-makers in African countries and what barriers, facilitators and outcomes exist for these strategies during policy formulation informed the initial selection of key terms. The need to capture KT literature relevant to health research and policy-making was balanced against the field’s large and overlapping nomenclature [14, 51]. Initial key terms included ‘knowledge translation’, ‘health researchers’ and ‘health policy-makers’. Final search terms were limited to those described by Graham et al. [12] and identified by McKibbon et al. [13] as highly sensitive for discriminating between KT and non-KT literature. These include ‘knowledge translation’, ‘knowledge transfer’, ‘knowledge exchange’, ‘research utilisation’, ‘implementation’, ‘dissemination’ and ‘diffusion’. These terms were supplemented with Medical Index Subject Headings and search filters for African countries [52]. Groups of terms were linked using the Boolean operator ‘AND’ then trialled in PubMed with the assistance of an experienced librarian. Following a preliminary search, evidence-based decision-making was added to this list to compliment search results. An additional file shows the final search strategy and list of key terms (Additional file 1).

Eligibility criteria were generated using the PICO (Population, Intervention, Comparator, Outcome) framework. Eligible populations included health researchers and public health policy-makers involved in management or in executive or policy-level decision-making about health programmes or services. Other knowledge users, such as non-government organisations, healthcare providers and patients, were excluded in line with the scope of the evidence map (focusing on policy formulation). Interventions included any activity designed to facilitate the use of research-based knowledge in public health policy-making. Both active KT strategies (for example, KT platforms, collaborative workshops) and passive KT strategies (including dissemination of reports or journal publications) were included. To increase the specificity of the map within a complex policy terrain, studies that focused on whole policy analysis and policy implementation processes were excluded, refining included studies to KT strategies employed before or during the policy formulation stage of the policy process. Any or no comparator between KT strategies were eligible for inclusion. Studies that identified KT strategies, barriers or facilitators, outcomes and contextual considerations were included. Outcomes of KT strategies included, but were not limited to, changes in knowledge, attitude, beliefs, behaviour, networks and partnerships. Papers were excluded if they focused mainly on theoretical and conceptual developments of KT, terminology debates and KT strategies employed in non-health related fields (for example, in economic, agricultural or criminal justice fields). Eligible study designs included randomised controlled trials, observational studies, surveys, qualitative research, case studies and mixed-methods research. Only primary empirical research was included. This excluded all analytical studies and research syntheses, such as systematic reviews. Since no synthesis studies were found that focused only on African countries, this was deemed an appropriate exclusion criterion. Multinational studies that involved African and non-African countries and met all other criteria were included if results could be traced to specific countries.

Four reference databases were searched using the advanced search tools in each database and the guidance of a second medical librarian. Databases included PubMed, CINAHL (via EbscoHost), Web of Science and Scopus. The search was limited to studies published in English between the years 2000 and 2017, inclusive, to capture modern KT strategies at work in African countries. Due to the large number of results obtained in PubMed, the ‘best match’ function was used to further limit search results for this database. The ‘best match’ function is based on a weighted frequency algorithm that enables only those studies with the highest frequency of targeted search terms to be included in search results. This strategy is based on the rationale that the risk of missing relevant articles is balanced against the logistical constraints of screening an overly burdensome number of irrelevant studies – which is a particular issue in large diverse topics such as this. Final search results were collated in EndNoteX8™ and supplemented via searches of Google Scholar and the Cochrane Library. A Dropbox database established opportunistically by the research team in September 2016 for the purposes of a broader research project was also included. This database contains a combination of relevant empirical studies and grey literature.

Following the removal of duplicates and preliminary screening of study titles and abstracts, full texts of remaining studies were procured, and a final screening was conducted using the established inclusion/exclusion criteria. In an iterative search process that promoted saturation, reference lists of final key texts were mined for additional relevant studies. Data was extracted on study author(s), title, year of publication, publication type, study design, location, underlying theory, funding source, sample size, participant populations, KT strategies employed, content focus and type of these strategies, reported barriers, facilitators, outcomes and contextual considerations. A searchable database containing this data was compiled using Microsoft Access. Frequency and thematic analysis were used to report study characteristics and to establish the final evidence map of KT strategies, barriers, facilitators and outcomes.

Initial themes and findings were verified during interviews with nine South African policy-makers and research managers during a subsequent companion study (reported elsewhere [53]), which provided substantiation of the evidence map, its main findings and tested the application of the evidence mapping approach in local health systems contexts.

## Results

### Search results

**Fig. 2:**
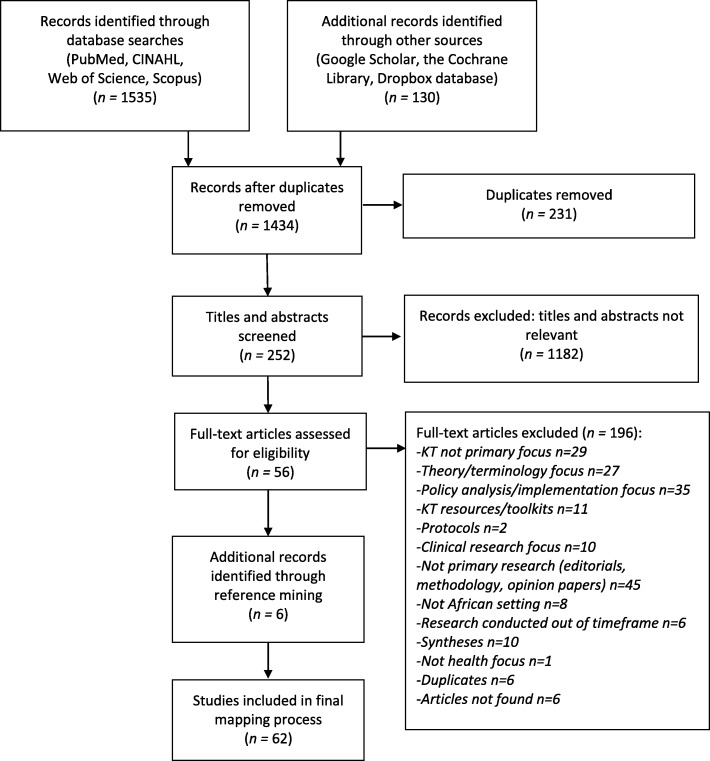
Study flow diagram. Adapted from Moher [102]

Initial search results identified a total of 1665 potential studies. Following removal of duplicates (*n =* 231) and screening of titles/abstract (*n =* 1182), the total number of studies was reduced to 252. Screening of full text items excluded an additional 196 studies. Reference mining of the final 56 studies presented an additional six studies for inclusion, which resulted in a total of 62 studies eligible for evidence mapping. Figure 2 provides the complete study flow diagram for this process.

### Study characteristics

**Fig. 3:**
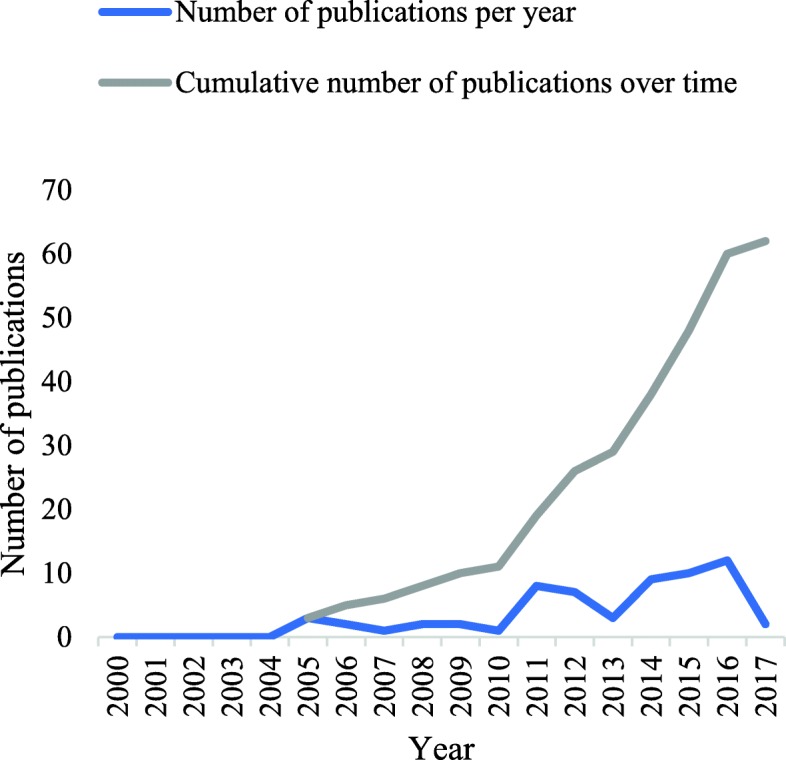
Number of publications by year

**Fig. 4:**
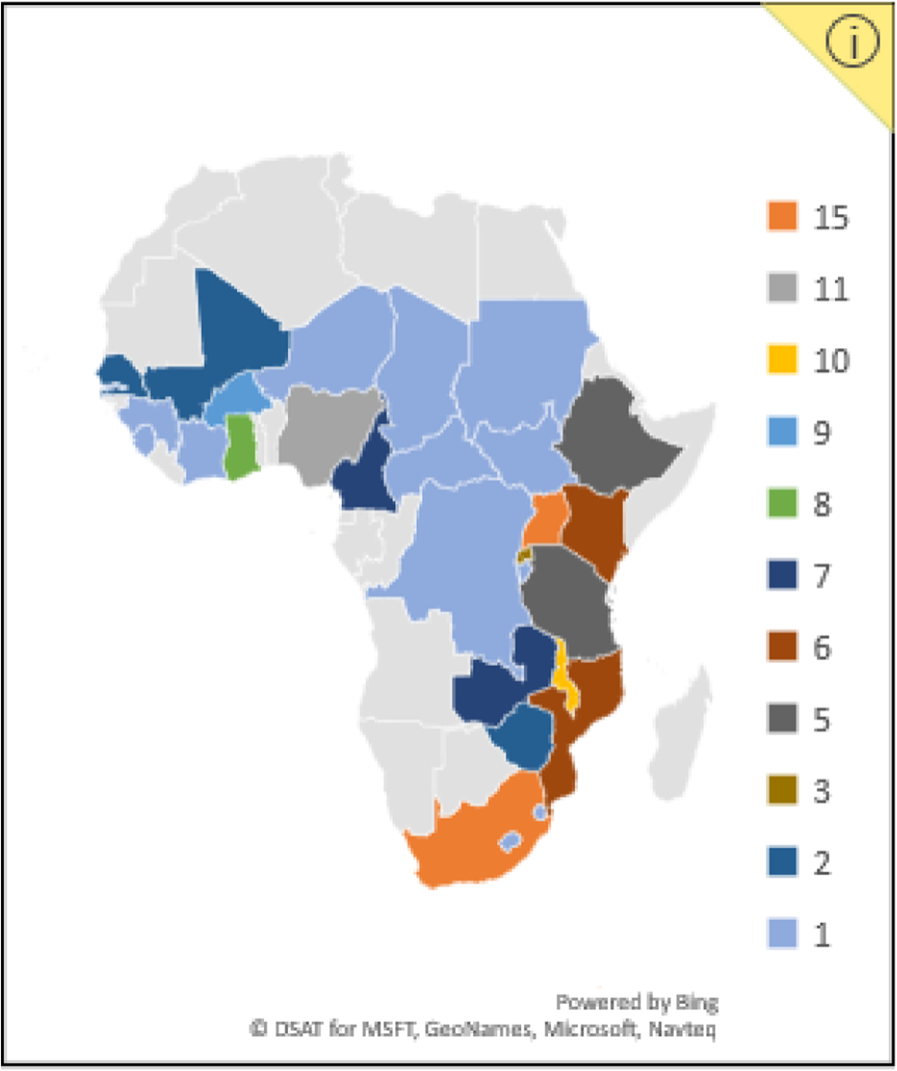
Distribution of publications by country

Included studies were published between 2005 and 2017, with a general increase in publications over time (Fig. 3). Studies were widely distributed across the African region, with most studies published in South Africa and Uganda (*n* = 15 studies each), followed by Nigeria (*n* =11) and Malawi (*n* = 10). Just over half (32/62) of the included studies were multinational, involving more than one African country, other LMICs, or a combination of upper-, lower- and middle-income countries. Figure 4 displays the geographical spread of included studies, as well as the large number of countries for which no research was found.

Overall, multiple case studies (of KT strategies) were the most popular study design, with 31 studies reporting its use. This was followed by qualitative designs (*n* = 12), mixed methods (*n* = 6), descriptive studies (*n* = 5), surveys (*n* = 3), modified ‘before and after’ study designs (*n* = 2), retrospective cohort designs (*n* = 1), social network analysis (*n* = 1) and structured reflection (*n* = 1). Funding for studies originated predominantly from international multilateral funders and foreign donor agencies (81%), with only six studies reporting local funding support as their primary source. Although a variety of content areas provided the backdrop for the focus on KT strategies, four broad areas accounted for over half of the included studies, namely maternal and child health (21%), governance, health information and systems issues (18%), malaria (10%), and HIV/AIDS (8%).

### KT strategies employed in African health systems

**Fig. 5:**
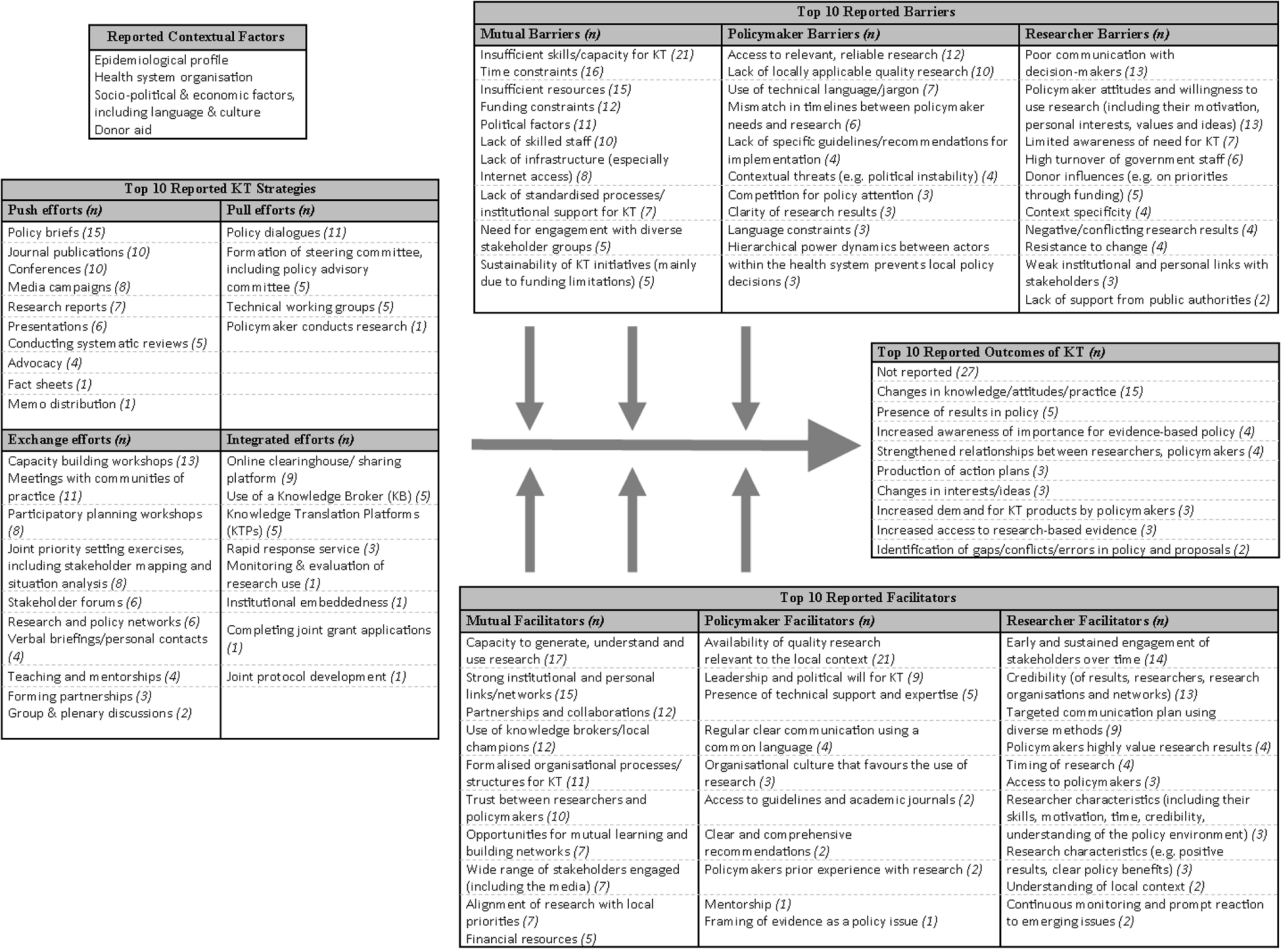
Summary map of knowledge translation (KT) strategies, influencing factors and outcomes. Note: arrows indicate direction of influence for factors on the KT process

Table 1 summarises the characteristics of KT strategies from an extract of four eligible studies. An additional file provides the same information for all 62 studies (Additional file 2). The commentary here reflects all included studies. Figure 5 maps the KT strategies, different influencing factors and outcomes found across all studies. The use of theory was reported in over half the studies (33/62) and varied widely from research utilisation models such as Weiss’ model of research use [54] to theories of planned behaviour (used to predict policy-makers’ intention to act on research evidence) [55]. Many studies reported the use of more than one theory, while no studies reported use of the same theory, reflecting the inherent heterogeneity in perspectives within the field [16].

**Table 1:** Characteristics of KT interventions (sample extract of four eligible studies)

Study author and date	Country	Underlying theory	KT intervention type^a^	KT strategies	Participants	Personnel	Reported contextual factors
Bennett et al. 2012 [103]	Multinational – Ghana, South Africa, Uganda	NR	Integrated efforts	Research reports and publications (indirect) Verbal briefings Policy briefs Conducting policy-relevant research and analysis Providing policy advice and technical assistance in policy formulation and evaluation Conducting policy dialogues at national level	Policy-makers, donors, NGOs	Members of research institutes	NR
Delany-Moretlwe et al. 2011 [104]	South Africa	NR	Exchange efforts	Building credibility through linkages – community advisory boards, community consultation workshop, monthly meetings Multiple means of communication – drama, music, radio and community eventsSMSFace-to-face meetings, telephonically, email (especially with policy-makers) Interdisciplinary workshops	Researchers	NR	Disease burden, health system organisation, socio-political context of South Africa
Guieu et al. 2016 [72]	Multinational – Kenya, Mozambique, South Africa, Burkina Faso, Malawi	NR	Exchange efforts	A ‘work package’ of translation activities, including strong involvement of stakeholders through inviting policy-makers to open meetings, key-informant interviews with policy-makers, workshops with policy-makers, fora for policy-maker feedback on research projects, policy advisory boards at each site, stakeholder workshops, policy recommendations in formats adapted to policy-maker needs	Researchers	NR	Importance of context noted, but not described,LMIC settings – resource constrained
Hennink and Stephenson 2005 [105]	Multinational – Malawi, Tanzania	Models of research utilisation –rational, incremental and political models	Push efforts	Workshops Research report distributed Academic channels (journals, conference presentations)	Health researchers, policy-makers and practitioners	NR	NR

^a^According to Lavis et al. [56] push–pull–integrated–exchange model

*NR* not reported

Using Lavis et al.’s [56] framework for linking research to action, studies were categorised as employing ‘push’, ‘pull,’ ‘integrated’ or ‘exchange’ type strategies for KT (see Box 2 for definitions of these strategies). Integrated and exchange strategies appeared most frequently, with 21 and 18 studies employing these, respectively, followed by push (*n* = 16) and pull strategies (*n* = 3).

Four studies did not report specific KT strategies, their focus on clarifying barriers and facilitators to the KT process.

Policy briefs (*n* = 15), capacity-building workshops (*n* = 13), policy dialogues (*n* = 11), and meetings between and within communities of practice (*n* = 11) were reported most frequently. Traditional strategies, such as conference presentations and journal publications, remain popular (*n* = 10 each), while the use of novel strategies, such as online clearinghouses/sharing platforms for research, media campaigns, knowledge translation platforms and knowledge brokers, also appear to be gaining ground. Knowledge translation platforms, specifically, demonstrate opportunities for the integrated use of KT strategies to foster collaboration and build capacity for research use. For example, with the assistance of the WHO’s Evidence-Informed Policy Network (EVIPNet), a knowledge translation platform in Malawi has been attributed with hosting stakeholder mapping exercises, capacity-building workshops and structured dialogues between national-level policy-makers, researchers and policy implementers as well as producing evidence briefs, facilitating the formation of a multidisciplinary steering committee and holding meetings between different communities of practice [57].

Despite clear information about who was targeted by KT strategies, few studies provided specific details about the duration, frequency or timing of events (27/62 reported this) or the personnel required to conduct activities (14/62 reported). In studies that did report this information, details varied significantly. In one study involving seven southern African countries, authors describe three week-long residential workshops facilitated by four researchers and include details on workshop duration and content [58]. While in another multinational study, a 3-year grant period is described in which activities occurred [11]. Similarly, reports of contextual factors were diverse, ranging from discussions of health system organisation, policy environment and epidemiological profile [59] to socio-political and economic contexts, including levels of decentralisation [60], legislative processes [61] and the influence of donor aid [62]. Only three studies reported cultural factors, all of which referred to issues of language [63–65]. Fourteen studies made no reference to contextual factors, while six noted these factors as important, but did not provide further details. Interestingly, despite the importance of contextual factors on the success of KT strategies, none of the reviewed studies established clear causal links between reported contextual factors and specific KT strategies or interventions, suggesting an urgent need for realist-type approaches to assessing KT strategies in complex local health systems.

### Barriers and facilitators of KT

A list of the ‘top 10’ barriers and facilitators reported across studies is provided in Fig. 5. Most studies reported both facilitators and barriers (*n* = 52), while a few reported only facilitators (*n* = 4) or only barriers (*n* = 5). One study did not report barriers or facilitators, but focused on the outcomes of implementing a health policy advisory committee as a KT platform in Nigeria [66]. A total of 46 different barriers and 46 facilitators were identified, and we further categorised these into those affecting policy-makers, researchers or both. Based on our analysis and ranking across the included studies, the main barriers affecting policy-makers include access to relevant, reliable research (*n* = 12) and a lack of locally applicable research (*n* = 10). For researchers, poor communication with policy decision-makers and policy-maker attitudes towards using research were reported as the most common barriers (*n* = 13 each), with limited awareness of the need for KT (*n* = 7) and a high turnover of government staff (*n* = 6) also being significant. There were several barriers that mutually affected researchers and policy-makers, such as insufficient skills and capacity to conduct KT activities (*n* = 21), time constraints (*n* = 16) and insufficient resources (*n* = 15). The most common resource constraint reported across the included studies was funding.

Regarding facilitators, the availability of quality research relevant to local contexts was reported by policy-makers as the greatest support to using research (*n* = 21). This was followed by consistent leadership and political will for KT (*n* = 9) and the presence of technical support and expertise (*n* = 5). Contrastingly, researchers were supported when they engaged with stakeholders early and sustained this engagement over time (*n* = 14). Credible research results, researchers and research organisations or networks were further noted to facilitate researcher efforts at KT (*n* = 13). Credibility is closely linked to trust between researchers and policy-makers and can serve as either a facilitator (*n* = 10) or a barrier (*n* = 2). Mutual facilitators once again highlight the importance of capacity to generate, understand and use research results (*n* = 17), demonstrating that the presence of KT skills is potentially as important as its absence. Furthermore, strong institutional links and networks (*n* = 15), partnerships and collaborations (*n* = 12), and the use of knowledge brokers or local champions (*n* = 12) highlight the significance of interactions between researchers and policy-makers in getting research to influence policy.

### Outcomes of KT

Figure 5 lists the main outcomes of KT strategies reported across studies. Twenty-seven studies (45%) did not report any outcomes for the KT strategies used. In the remaining 35 studies, there was little overlap and most outcomes appeared to be study specific. For example, one study focused on the activity output of a knowledge broker specifically employed for KT [65], while others reported the creation of a research repository [45] and a database of researchers and policy-makers [67] as direct outcomes of KT processes. The most frequently reported outcomes were changes in knowledge, attitudes and practices (*n* = 15) such as improved understanding of the health policy-making process [68]. Studies that attempted to categorise outcomes employed three types of knowledge use, namely symbolic use, conceptual use and instrumental use. These types were reported in nine studies, in which instrumental and conceptual use was most commonly reported. For example, in one South African case study [69], researchers found that the instrumental use of research directly contributed to the development of a model on primary healthcare service delivery while conceptual use helped place mental health issues on the policy agenda. A study in Burkina Faso described the practical changes in behaviour (instrumental use) and the new knowledge acquired (conceptual use) by participants in a 2-day workshop on malaria [70]. Only one study compared the extent to which different uses of research were employed, noting that instrumental use occurred the least across six LMIC countries’ analysed policies and programmes [71]. The large number of studies neglecting to report study outcomes (including knowledge use), and the heterogeneous outcomes in those that did, reflect persisting difficulties in the identification and reporting of KT outcomes, an issue that has been noted previously [14, 36, 37]. Three studies noted this difficulty specifically, recognising the often unclear and long-term impacts of KT strategies on research use [71–73].

## Discussion

This evidence mapping exercise has sought to impose some framing on what is known to be a large, diverse and dispersed body of research and literature. The literature on KT in African countries (as defined in this mapping exercise) is widely distributed and growing steadily. However, significant disparities exist between countries, and many remain without any significant published evidence of research related to KT (Fig. 4). This highlights an ongoing need to boost local research capacities on KT practices in many African countries [1, 74]. High levels of foreign donor funding (80% of studies in this case) create opportunities for conducting local research, particularly in resource-constrained settings. However, the extent to which this is sustainable and allows local researchers and policy-makers to direct the research agenda is debatable [75].

Together with Uganda, South Africa demonstrates a relatively established research base compared to other African nations, ranking the highest generators of KT research on the continent. In South Africa, the presence of historically strong research institutions that house platforms for KT is a likely contributor to this base. For example, the Centre for Evidence-based Healthcare at Stellenbosch University and the Knowledge Translation Unit at the University of Cape Town’s Lung Institute both actively target the production, synthesis and use of health research in policy and practice [76, 77]. The Cochrane African Network is another KT platform housed in the South African Medical Research Council, a nationally funded research organisation [78]. These institutions provide a rich support system for KT in the country. However, despite local and international efforts, even in countries such as South Africa or Uganda, the research conducted to date is by no means sufficient to meet the demands of diverse African contexts, and there remains an “*inadequate evidence base for doing evidence-based KT*” in health policy ([79], p. 729).

Filling this research gap requires consideration of factors beyond increasing research quantity across geographical areas. A central barrier identified by this mapping process was the lack of high-quality evidence relevant to local health systems contexts. Although context specificity is a key challenge to KT research production and use, research that meets local demands and aligns with local priorities is more likely to be translated into policy [3, 26, 80]. Capacity-building initiatives that target the development of KT skills for local researchers and health policy-makers will be an essential component to improving the availability and applicability of research going forward.

Furthermore, to develop KT research quality, study designs that extend beyond case studies and descriptive work should be encouraged. Realist approaches, pragmatic trials, impact evaluations (of KT strategies), implementation research and participatory action research are thought to hold particular promise for developing, assessing and implementing the social and contextually sensitive interventions inherently associated with KT [20, 81–84]. These designs offer opportunities for increased scientific rigour and better consideration of contextual differences, including knowledge and power imbalances, to understand what works, for whom and under what circumstances.

The diverse pool of KT strategies identified in this mapping review demonstrate a variety of options available to researchers and policy-makers when attempting to ‘do’ KT in African settings. The combined use of policy briefs, workshops, policy dialogues and meetings with communities of practice hold particular promise, as these are widely used and likely familiar to both researchers and policy-makers. However, the time, effort and resources involved in these activities should not be underestimated. Conducting KT requires investment if research-based knowledge is to be communicated appropriately and with the best chance of influencing its intended audience [85]. The low level of locally generated funding for KT research found in this review highlights the need to advocate for stronger national funding mechanisms to support a more sustainable model of knowledge production and use.

Furthermore, this review highlights the increasing preference for and value of integrated and exchange type strategies. An integrated KT approach recognises that, to influence policy, audiences need to be targeted early and in multiple ways, leveraging personal and professional networks that facilitate the bidirectional flow of knowledge [2, 30, 86]. Co-produced knowledge based on the collaborative efforts of both researchers and policy-makers at all stages of the research process, including research generation, is a core tenet in this approach and has the added benefit of addressing policy-makers’ need for more locally relevant, reliable research. This is in line with findings from upper-income countries that emphasise the need for integrated KT efforts that promote early and sustained engagements between stakeholders [2, 86–90].

The diversity in strategies found may be appealing as it reflects options that can be shaped to different contexts. However, this diversity has been criticised for demonstrating uncoordinated, haphazard and fragmented efforts to narrow the knowledge gap in Africa [91]. The wide variations in theories and outcomes identified here support this conclusion and contribute to a persistently unclear change pathway between research, KT strategies and policy formulation. Furthermore, the lack of uniformity and clarity on KT outcomes makes it almost impossible to compare different interventions or to measure progress (locally or regionally). There is an urgent need to improve theories and methods that rigoously assess KT interventions in real time and identify relevant outcomes that are sensitive to the short- and long-term effects of KT activities. This issue is not unique to African contexts and reflects concerns expressed in the global literature [7, 14, 20, 36–38, 92–95].

A final point arising from this mapping process concerns issues of capacity. Knowledge and skills in prioritising, generating, synthesising and applying research were identified as the most common KT barriers and facilitators for both researchers and policy-makers. Capacity constraints have also been identified as one cause of the know–do gap and a key priority for addressing health in LMICs [1]. To date, individual capacity has largely been the focus of efforts to improve research production and use in policy. However, recognising the need for broader system strengthening, tools that focus on improving institutional capacity for research use are on the rise [27, 34, 96]. Additionally, regional and international partnerships are supporting many countries to develop capacity to analyse their own health systems and develop locally appropriate KT strategies such as, for example, EVIPNet in Malawi and Uganda [57, 97], the West African Health Organization in Burkina Faso, Nigeria, Senegal and Sierra Leone [98], and the Consortium for Health Policy and Systems Analysis in Africa in South Africa, Tanzania, Ghana, Kenya and Nigeria [99]. These capacity-building efforts serve as useful learning opportunities for other settings, including how to strengthen local research and policy communities, promote collaboration, encourage the formation of KT support networks and the more equitable distribution of knowledge.

### Limitations and implications for evidence map use

The broad search criteria and diverse search terms employed in constructing this evidence map reflect the complexity of KT. While the final set of included studies indicate the breadth of research on the African continent, there is a need to refine these criteria and appraise the quality of resulting studies in a more detailed systematic review. A potential focus for further research may be to trace KT strategies, outcomes, barriers and facilitators through specific countries to provide more contextualised theories of change [100].

Furthermore, time and resource constraints precluded a comprehensive search of the literature. Few databases and online searches of key sites mean important studies may have been omitted from the final list of studies. Expanded searches of the grey literature and inclusion of additional databases should complement and enhance the current map. However, given the scoping nature of the evidence map, this was deemed appropriate for this study.

Finally, an evidence map is a cross-section of studies conducted within a specific timeframe. The result is a snapshot of KT strategies and their outcomes, barriers and facilitators. The dynamic influence of contexts on KT interventions, especially in Africa, mean this map should serve only as a starting point. A detailed contextual analysis prior to its application and use is recommended.

## Conclusion

This review presents an overview of the literature on KT in African health systems by mapping the strategies, outcomes, barriers and facilitators at work across different countries. In doing so, it provides a useful summary for health policy-makers and researchers seeking to conduct KT activities in local contexts. Additionally, it highlights important evidence gaps, including the need for (1) increased research on KT strategies, barriers, facilitators and outcomes that includes a greater geographical scope, both within and across national borders; (2) greater variety in research approaches to KT, including more realist-type and evaluative designs and increased emphasis on contextual descriptions and outcomes of KT interventions; (3) exploration into the impact of donor funding on KT and its outcomes; (4) further testing, development and application of theoretical frameworks that enhance understanding of KT in African settings; (5) exploration into the impact of an integrated KT approach that fosters engagement and collaboration between researchers and policy-makers throughout (and beyond) the research process; and (6) initiatives that build individual and organisational capacity to generate, understand and use research.

This evidence mapping study also confirms the usefulness of this type of systematised review approach for navigation of dispersed and diverse research terrains, where there is little consensus or definitional clarity. Further evidence mapping activities would be useful on more specific KT issues, and in more context-specific settings, so that researching policy-makers and policy-influencing researchers have more readily available and suitable evidence to better inform their practice. Understanding and developing learning health systems is becoming a major focus of the global health policy and systems community [101], and localised and focused evidence maps would be a useful tool towards this practice.

Box 1 Current challenges to knowledge translation (KT) in low- and middle-income country health systems • Access to good quality relevant research

• Unknown effectiveness/impact of KT strategies

• Difficulty identifying clear outcomes of KT activities

• Need to strengthen valid instruments to measure KT

• Complex nature of KT including its power dynamics, differing timelines and unique contexts

• Unclear choice of KT strategies as they vary by context

• Complex systems issues, including historically weak relationship between health and research systems

• Limited local funding and research capacity

Box 2 Push, pull, exchange and integrated models of knowledge translation [56] • ‘Push’ strategies are led by researchers, intermediary groups or other purveyors of research, and typically involve providing information to research users.

• User ‘pull’ efforts are led by research users who request information and/or research evidence based on their needs.

• ‘Exchange’ efforts rely on partnerships between researchers and research users who collaborate over short- or long-term processes for mutual benefit.

• ‘Integrated’ strategies include elements of push, pull and exchange approaches in large-scale knowledge translation platforms that work to connect policy needs with research tools.

## Additional files

Additional file 1: List of databases, final search strategies and key terms. (DOCX 13 kb)

Additional file 2: Characteristics of knowledge translation (KT) interventions (all studies). (DOCX 43 kb)

## Abbreviations

EVIPNet: Evidence-Informed Policy Network
KT: knowledge translation
LMICs: low- and middle-income countries
