# Supplementary material for: Evidence map of knowledge translation strategies, outcomes, facilitators and barriers in African health systems
Source: Health Res Policy Syst. 2019 Feb 7;17:16. doi: 10.1186/s12961-019-0419-0 (PMC6367796; doi:10.1186/s12961-019-0419-0)
Supplement: Supplementary file 1 — List of databases, final search strategies and key terms. (DOCX 13 kb) [file 12961_2019_419_MOESM1_ESM.docx]

**Additional file 1: List of databases, final search strategies and key terms**

| **PUBMED** | **CINAHL (via EBSCOhost)**  **Web of Science**  **Scopus**  **Cochrane Library** |
| --- | --- |
| knowledge translation OR knowledge transfer OR knowledge exchange OR research utilization OR research utilisation OR implementation OR dissemination OR diffusion OR evidence-based decision making OR Translational Medical Research  AND  Policy Making OR Health Policy OR policy makers OR policy making OR health policy OR health researchers  AND  Africa OR African OR Algeria OR Angola OR Benin OR Botswana OR Burkina Faso OR Burundi OR Cameroon OR Canary Islands OR Cape Verde OR Central African Republic OR Chad OR Comoros OR Congo OR Democratic Republic of Congo OR Djibouti OR Egypt OR Eritrea OR Ethiopia OR Gabon OR Gambia OR Ghana OR Guinea OR Ivory Coast OR Cote d'Ivoire OR Jamahiriya OR Kenya OR Lesotho OR Liberia OR Libya OR Madagascar OR Malawi OR Mali OR Mauritania OR Mauritius OR Mayotte OR Morocco OR Mozambique OR Namibia OR Niger OR Nigeria OR Principe OR Reunion OR Rwanda OR Sao Tome OR Senegal OR Seychelles OR Sierra Leone OR Somalia OR St Helena OR Sudan OR Swaziland OR Tanzania OR Togo OR Tunisia OR Uganda OR Western Sahara OR Zaire OR Zambia OR Zimbabwe | “knowledge translation” OR “knowledge transfer” OR “knowledge exchange” OR “research utilization” OR “research utilisation” OR implementation OR dissemination OR diffusion OR “evidence-based decision making” OR “Translational Medical Research”  AND  “Policy Making” OR “Health Policy” OR “policy makers” OR “policy making” OR “health policy” OR “health researchers”  AND  Africa OR African OR Algeria OR Angola OR Benin OR Botswana OR Burkina Faso OR Burundi OR Cameroon OR “Canary Islands” OR “Cape Verde” OR “Central African Republic” OR Chad OR Comoros OR Congo OR “Democratic Republic of Congo” OR Djibouti OR Egypt OR Eritrea OR Ethiopia OR Gabon OR Gambia OR Ghana OR Guinea OR “Ivory Coast” OR “Cote d'Ivoire” OR Jamahiriya OR Kenya OR Lesotho OR Liberia OR Libya OR Madagascar OR Malawi OR Mali OR Mauritania OR Mauritius OR Mayotte OR Morocco OR Mozambique OR Namibia OR Niger OR Nigeria OR Principe OR Reunion OR Rwanda OR “Sao Tome” OR Senegal OR Seychelles OR “Sierra Leone” OR Somalia OR “St Helena” OR Sudan OR Swaziland OR Tanzania OR Togo OR Tunisia OR Uganda OR “Western Sahara” OR Zaire OR Zambia OR Zimbabwe |
