# Supplementary material for: Evidence map of knowledge translation strategies, outcomes, facilitators and barriers in African health systems
Source: Health Res Policy Syst. 2019 Feb 7;17:16. doi: 10.1186/s12961-019-0419-0 (PMC6367796; doi:10.1186/s12961-019-0419-0)
Supplement: Supplementary file 2 — Characteristics of knowledge translation (KT) interventions (all studies). (DOCX 43 kb) [file 12961_2019_419_MOESM2_ESM.docx]

**Additional file 2: Characteristics of KT interventions (all studies)**

| **Study** | **Country** | **Underlying Theory** | **KT Type** | **KT Strategies** | **Participants** | **Personnel** | **Reported Contextual Factors** |
| --- | --- | --- | --- | --- | --- | --- | --- |
| Aaserud *et al.* 2005 | Multinational - Rwanda, South Africa | NR | Integrated Efforts | Small group discussions Plenary discussions Journal article publication Mass media campaigns | Health authorities (local, national, global), professional organisations, civil society, drug licencing agency, pharmaceutical industry, mass media, influential professionals | NR | NR |
| Albert *et al.* 2007 | Mali | Giorgi's phenomenological approach | Push Efforts | Reports | National policymakers | NR | As a "verbal society" many policy-makers prefer verbal reports to documentation. |
| Ashford *et al.* 2006 | Kenya | Interactive model | Integrated Efforts | District seminars Region-specific presentations District fact sheets Press alerts/Media release Collaborative regional planning seminars to develop district-level work plans Multidisciplinary working groups | District medical health officers, nurses, health educators, NGO representatives, private physicians, hospital representatives, and personnel from the two government ministries (health and planning) | Officials from Ministry of Health & Ministry of Planning | Kenyan government’s new decentralized programme-reform initiative. |
| Avan *et al.* 2016 | Multinational - Ethiopia, Nigeria | TELOS framework | Push Efforts | District data-sharing platform Coordinating & synthesizing local programme health data | State and district level administrators, NGO representatives, primary and secondary care clinical staff | NR | Culture of democratic governance, decentralization, public–private partnerships. |
| Ayah *et al.* 2014 | Multinational - Uganda, DRC, Tanzania, Kenya, Rwanda, Ethiopia | NR | Push Efforts | Dissemination at scientific conferences and workshops | Academic researchers | NR | Limited press freedom in some countries. |
| Beesley *et al.* 2011 | South Sudan | Walt and Gilson policy analysis triangle | Push Efforts | Consultative workshop | NGO and Ministry of Health workers | National Ministry of Health staff | Post-conflict environment (poor quality of care, inefficiency, heterogeneous standards, low and uneven coverage stood out as the defining features of a fragmented health space). Detailed description according to framework. |
| Behague *et al.* 2009 | Multinational - Burkina Faso, Ghana, Malawi | Network theory | Exchange Efforts | Multi-institutional consortium | Opinion leaders, policymakers, researchers, health administrators, clinicians | NR | Political contexts promote uniformity of methodology and policy approaches. Donor Priorities and distribution of limited resources shape interpretation of research findings. |
| Bennett *et al.* 2012 | Multinational - Ghana, South Africa, Uganda | NR | Integrated Efforts | Research reports and publications (indirect) Verbal briefings Policy briefs Conducting policy-relevant research and analysis Providing policy advice and technical assistance in policy formulation and evaluation Conducting policy dialogues at national level | Policymakers, donors, NGO | Members of research institutes | NR |
| Beran *et al.* 2015 | Multinational - Mali, Mozambique, Zambia | NR | Push Efforts | Rapid assessment tool  Reports Publications | National policymakers, researchers | NR | NR |
| Berman *et al.* 2015 | Malawi | NR | Integrated Efforts | Stakeholder mapping exercise  Initial capacity building workshops Evidence briefs Structured deliberative dialogues Formation of multidisciplinary steering committee Structured prioritization process Meetings with Communities of Practice | National-level policymakers, researchers and implementers | Researchers and policymakers | NR |
| Blau *et al.* 2012 | Cote d'Ivoire | NR | Pull Efforts | Technical advisory group Meetings with national health authorities and partners Development of concept paper National workshop Appointed committee Annual consultations | National experts, Ministry of Health representatives, partner representatives | NR | Detailed demographics and disease profile, national vaccine schedules, coverage rates over time, current health system issues, national training capacity (universities), socio-political context. |
| Cole *et al.* 2016 | Malawi | NR | Push Efforts | National and institutional meetings Sponsoring attendance at conferences Close relationships with individuals in the print media Sponsorship of an issue of the Malawi Medical Journal Meetings of special interest groups  Interactive website  National and international conference presentations  Publication in international journals | International funders, national research users, researchers, consultants | NR | Research system development over time, key actors, health system history and development, socio-political history. |
| Dagenais *et al.* 2013 | Burkina Faso | NR | Push Efforts | Policy briefs District/regional workshops Scientific communication/publications Theatre forums Cultural workshops Advocacy publications Press conference | NGO, policymakers, technical partners, civil society, lay health workers | NR | NR |
| Dagenais *et al.* 2015 | Burkina Faso | Grounded theory, Logic model developed for KB process | Integrated Efforts | Participatory planning workshops Collaborative training workshops Intensive KB recruitment and training | Canadian and African researchers, a knowledge broker, health practitioners, and policymakers | NR | French speaking nation, primary disease burden, centralised decision-making, high level of donor aid. |
| Daniels and Lewin 2008 | South Africa | Kingdon’s policy agenda setting; Influencers of the policy process (political science framework) | Exchange Efforts | Policy networks | Policymakers, academic clinicians | NR | Democratic change, health system reform, policy context equals a window of opportunity, burden of problem/disease. |
| Daniels and Lewin 2011 | South Africa | NR | Exchange Efforts | Professional networks and links  Meetings Personal contact Advocacy Local conferences | Local researchers and policymakers | NR | Detailed local and international context description of medical advancements in the field, political changes over time in South Africa. |
| de Carvalho *et al.* 2015 | Ghana | WHO KT framework on ageing and health | Integrated Efforts | Joint priority setting Local evidence collection Policy Dialogue  Policy briefs  Presentations | Representatives from Ghana health service, teaching hospitals, professional bodies, WHO, HelpAge Ghana | Ghana Health Service, WHO | Well-structured health system, adaptable to meet needs of ageing population; policy in place and favourable political environment; good local data; epidemiological transition. |
| Delany-Moretlwe *et al.* 2011 | South Africa | NR | Exchange Efforts | Building credibility through linkages - Community advisory boards, community consultation workshop, monthly meetings Multiple means of communication - drama, music, radio and community events SMS Face-to-face meetings, telephonically, email (especially with policymakers) Interdisciplinary workshops | Researchers | NR | Misunderstandings, mistrust between researchers, policymakers and society; disease profile, health system and political context of SA. |
| El-Jardali *et al.* 2014 | Multinational - Nigeria, Burkina Faso, Cameroon, Central African Republic, Ethiopia, Uganda, Sudan, Zambia | Linking research to action framework | Integrated Efforts | Knowledge translation platforms (KTPs) - Deliberative dialogues informed by evidence briefs - Capacity building workshops - Rapid response services - Online clearinghouses - Assess and enhance the capacity of research users | Policymakers, stakeholders and KTP leaders | NR | NR |
| Fiankor and Akussah 2012 | Ghana | NR | Pull Efforts | Media (newspapers, radio, TV) Meetings  Conferences  Seminars  Workshops Government publications Reports Published and unpublished materials Other (personal contacts, conversation, gossip and advice) | Assembly policymakers | NR | Local government system. |
| Guieu *et al.* 2016 | Multinational - Kenya, Mozambique, South Africa, Burkina Faso, Malawi | NR | Exchange Efforts | A "work package" of translation activities, including strong involvement of stakeholders through: - Inviting policymakers to open meetings - Key-informant interviews with policymakers - Workshops with policymakers - Fora for policymaker feedback on research projects - Policy advisory boards at each site - Stakeholder workshops  - Policy recommendations in format adapted to policymaker needs | Researchers | NR | Importance of context noted, but not described; LMIC setting - resource constrained. |
| Hawkes *et al.* 2016 | Multinational - Gambia, Nigeria | Department of International Development capacity framework-individual, organizational, institutional levels | Integrated Efforts | Pre-intervention situation analysis Access to research through infrastructure and online platforms Regular meetings Training workshops with policymakers and managers Training health journalists Biannual policy retreats Established a Health Policy-Research Committee | Researchers, policymakers, managers | NR | Cross country variations noted, but not explicit; importance of political context noted. |
| Hennink and Stephenson 2005 | Multinational - Malawi, Tanzania | Models of research utilization - rational, incremental and political models | Push Efforts | Workshops Research report distributed Academic channels (journals, conference presentations) | Health researchers, policymakers, and practitioners | NR | NR |
| Hunsmann 2012 | Tanzania | Grounded theory approach; Political economy perspective | None | NR | Health officials, donor representatives, academic researchers, NGO consultants | NR | Strongly heteronomous and most implementing organisations heavily depend on international funding. |
| Hutchinson *et al.* 2011 | Multinational - Malawi, Uganda, Zambia | ODI framework - context, evidence, links | None | Dissemination Advocacy | NR | NR | Political and economic context influenced interpretation of results. |
| Hyder *et al.* 2011 | Multinational - Malawi, Egypt | NR | None | NR | National-level policymakers | NR | Political context factors: legislative processes, parliamentary machinery and budgetary policies, electoral impact. |
| Keita *et al.* 2017 | Multinational - Burkina Faso, Nigeria, Senegal and Sierra Leone | ‘Doing by learning’ approach | Exchange Efforts | Steering committees Formal meetings Regular telephone and email exchanges | Researchers, stakeholders, decision-makers | NR | Country specific factors alluded to, but not explicit. |
| Kok *et al.* 2016 | Ghana | Actor-scenario perspective | Exchange Efforts | Discussions Knowledge brokers ("user-investigator") Research reports Memo Discussion forum Working groups | Researchers, policymakers | NR | NR |
| Koon *et al.* 2012 | Multinational - Nigeria, Cameroon | WHO building blocks; Conceptual framework of institutional embeddedness | Integrated Efforts | Institutional embeddedness | High-ranking researchers, national policymakers | NR | Country specific noted, but not explicit. |
| Kwamie and Nabyonga-Orem 2016 | Multinational - Guinea, Chad | Realist framework: context-mechanism-outcome | Exchange Efforts | Policy dialogue | Policymakers, civil society, development partners | NR | Socio-political factors, maternal and under-five mortality rates, hierarchical health system organisation. |
| Lairumbi *et al.* 2008 | Kenya | NR | Push Efforts | Publication in journals Presentation in workshops/ seminars/conferences Presentation of brief reports Project steering committees involving policymakers Teaching at universities | Policymakers, policy implementers, researchers, health advisors | NR | Health system context, organisational arrangements. |
| Langlois *et al.* 2016 | Multinational -South Africa, Cameroon | NR | Integrated Efforts | Baseline situational analysis Capacity building workshops Subsequent meetings and dialogues Online sharing platform (“Ezcollab”) Systematic reviews | Subnational policymakers | Health field experts (KT buddy) | Level of government centralization. |
| Lavis *et al.* 2010 | Multinational - Ghana, Tanzania, Senegal | NR | Integrated Efforts | Systematic reviews Access to a searchable database of research products Long-term partnerships | Researchers | NR | NR |
| Mbonye and Magnussen 2013 | Uganda | NR | Exchange Efforts | Workshops Presentations | Midlevel policymakers, researchers, media | NR | Country's high Burden of Disease (BoD), decentralised health system organisation. |
| Mc Sween-Cadieux *et al.* 2017 | Burkina Faso | NR | Push Efforts | Workshops Policy briefs Local knowledge broker | Researchers, policymakers, programme managers, NGOs, health professionals, civil society organisations | Researchers | Malaria policy history. |
| Mijumbi *et al.* 2014 | Uganda | NR | Pull Efforts | Question clarification Research synthesis Written evidence brief | Policymakers and stakeholders, including technical support staff, health managers, advocacy personnel, development partners | NR | NR |
| Mirzoev *et al.* 2012 | Multinational - Ghana, South Africa, Uganda, Zambia | Conceptual framework - study specific | Exchange Efforts | Research-policy partnerships | Ministries of Health and research organisations | NR | Detailed: healthcare expenditure, form of health system decentralisation, mental health worker distribution, status of current mental health policy. |
| Moat *et al.* 2014 | Multinational - Burkina Faso, Cameroon, Ethiopia, Nigeria, Uganda, Zambia | Theory of planned behaviour | Integrated Efforts | Evidence briefs Deliberative dialogues | Policymakers, stakeholders, researchers | NR | Role of political context noted, but not specific. |
| Mwendera *et al.* 2016 | Malawi | Conceptual framework based on Ottawa Model of Research Use (OMRU) | Exchange Efforts | Knowledge translation platforms Technical working groups Systematic reviews Policy briefs Teaching evidence-based healthcare Annual research dissemination conferences | Researchers, policymakers, programme managers and key stakeholders | NR | National Malaria policy and BoD. |
| Nabudere *et al.* 2013 | Uganda | SUPPORT Tools framework | Exchange Efforts | Policy brief Stakeholder dialogue meetings | Policymakers, researchers | NR | Maternal health statistics, health service delivery. |
| Nabyonga-Orem *et al.* 2014a | Uganda | Middle range theory (MRT), policy development framework | Integrated Efforts | Media - newspaper reports Partnership forums, including:  - Malaria Case Management Technical Working Group (MCMWG)  - The Interagency Coordination Committee for Malaria  - The national stakeholder forum | Researchers, policymakers, civil society, service providers, media | NR | Malaria treatment history in region, previous policy timeline. |
| Nabyonga-Orem *et al.* 2014b | Uganda | Middle range theory (MRT) | None | Dissemination tools: Summary reports Policy briefs Once-off KT task force | Donors, policymakers, researchers, civil society, journalist, private service provider | NR | Polarisation of stakeholders on issue, detailed timeline of political events. |
| Naude *et al.* 2015 | Multinational- South Africa, Cameroon | Consolidated Framework for Implementation Research (CFIR) | Exchange Efforts | NA | Subnational policymakers (provincial and regional) | NR | Constitutional democracies, country statistics. |
| Oliver and Dickson 2016 | Multinational – African region | Overlapping social worlds - conceptual framework | Push Efforts | Systematic reviews | Policymakers, systematic reviewers | NR | NR |
| Ongolo-Zogo *et al.* 2014 | Multinational - Cameroon, Uganda | Combined frameworks of knowledge brokerage and the integrated model for knowledge translation | Integrated Efforts | Knowledge translation platforms Evidence briefs  Policy dialogues Rapid evidence syntheses Online clearinghouse Capacity building workshops Advocacy meetings Presentations Stakeholder and research mapping Priority setting exercises Grant applications | Policymakers, researchers and other stakeholders | NR | Health system, political, social and economic context in each country, MDG indicators, historical account. |
| Onwujekwe *et al.* 2015 | Nigeria | Conceptual framework for assessing the role of evidence in policy development | Integrated Efforts | Reports Publications Expert consultation meetings International documents | Policymakers, researchers, civil society, professional groups, development partners, health workers | NR | Detailed socio-political, health system context for each policy case. |
| Pittman 2006 | Multinational - South Africa | NR | Push Efforts | Crafting and selecting messengers in accordance with audience’s concerns. Delaying publication until interactions with policy makers had occurred. Monitoring public opinion. Ideas relating to the style and content of government- sponsored commission reports. | Researchers and policymakers | NR | Democratic changes, allegations of corruption, AIDS denialism, public outcry, confrontational environment and political controversy. |
| Rehfuess *et al.* 2016 | Multinational- Burundi, Ethiopia, Malawi, Rwanda, South Africa, Uganda | NR | Exchange Efforts | Structured participatory approach: - Online survey of policymakers and partners (gathering relevant information) - Iterative face-to-face consultations through conferences, discussions - Identify gaps in evidence base through evidence maps - Joint protocol development through cross-national research teams | Researchers, high level policymakers | NR | NR |
| Rispel and Doherty 2011 | South Africa | NR | Integrated Efforts | University-based research unit involved in: - Meetings - Workshops - Research reports - Journal articles - Conference participation - Policy briefs - Facilitating stakeholder engagement - Media engagement (least amount) | Researchers, policymakers, Centre for Health Policy stakeholders | NR | Socio-political history of South Africa, health care reform. |
| Rodríguez *et al.* 2015 | Multinational- Niger, Kenya and Mozambique | Carol Weiss’ models of research utilization; Walt and Gilson policy triangle | Push Efforts | Regional meetings Scientific journal series in Lancet Evaluation reports Knowledge brokers | National policymakers, donors, researchers, civil society | NR | Country specific - disease burden, health service distribution. |
| Rosenbaum *et al.* 2011 | Multinational- South Africa, Uganda | NR | Push Efforts | Evidence summaries of systematic reviews | Policymakers | NR | NR |
| Shearer *et al.* 2014 | Burkina Faso | Social networking analysis | Exchange Efforts | Interpersonal relationships | Policy actors | NR | Political, health system organisation. |
| Shroff *et al.* 2015 | Multinational- Cameroon, Nigeria, Zambia | Jacobson et al. KT framework | Integrated Efforts | Policy briefs Policy dialogue Formal research to action group (Zambia) & Health Policy Advisory Committee (Nigeria)  Researcher directory Training workshops Online clearinghouse of policy briefs & relevant academic literature Radio programmes (Nigeria) | National policymakers, researchers | NR | Commented on influence of context, but not explicit. |
| Ssengooba *et al.* 2011 | Uganda | Multiple KT frameworks | Integrated Efforts | Decision-making fora (e.g. national advisory committees) Policy briefings Mass media | Researchers, policymakers, media practitioners | NR | BoD, health system design, MDG progress, local research environment. |
| Stewart *et al.* 2005 | Multinational- Zambia, Zimbabwe, South Africa, Tanzania, Swaziland, Lesotho and Mozambique | NR | Exchange Efforts | Mixed and participatory residential training workshops in accessing and appraising research Creation of informal networks | Policymakers, practitioners, researchers | Four researchers | Political and economic factors. |
| Uneke *et al.* 2015a | Nigeria | NR | Integrated Efforts | Training workshops Mentoring Policy briefs | Health policymakers | NR | Burden of disease. |
| Uneke *et al.* 2012 | Nigeria | NR | Exchange Efforts | Training workshop | Researchers, policymakers, health stakeholders | NR | NR |
| Uneke *et al.* 2015b | Nigeria | Implementation research framework | Integrated Efforts | Knowledge translation platform: - Capacity building training workshops and university short courses - Mentorships - Policy briefs - Multi-stakeholder policy dialogue | Policymakers, researchers | NR | History of platform development. |
| Uzochukwu *et al.* 2016 | Nigeria | NR | Exchange Efforts | Researcher-policymaker engagement via 4 strategies: 1. Policymakers and stakeholders seeking evidence from researchers 2. Involving stakeholders in designing objectives of a research and throughout the research period 3. Facilitating policymaker research | Researchers and policymakers | NR | Influence of political context noted, but not specific. |
| Vargas *et al.* 2016 | Multinational - Ghana, Malawi, Mozambique | Instrumental, conceptual, symbolic use framework | Push Efforts | Executive summaries Policy briefs Bulletins Web pages Scientific papers | Policymakers and program managers | NR | Country specific - organisations for evidence-based health policy described only. |
| Woelk *et al.* 2009 | Multinational - Mozambique, South Africa, Zimbabwe | NR | Integrated Efforts | Lobby groups Champions Research and policy networks | Health officials, policymakers, researchers, donors | NR | Noted importance of specific political and economic contextual factors. |
| Zachariah *et al.* 2014 | Multinational- Kenya | NR | Push Efforts | Training workshops | Public health practitioners | NR | NR |
